# Increased Expression of Plasma miRNA-320a and let-7b-5p in Heroin-Dependent Patients and Its Clinical Significance

**DOI:** 10.3389/fpsyt.2021.679206

**Published:** 2021-06-29

**Authors:** Haixiong Liu, Wenjin Xu, Jiying Feng, Hong Ma, Jianbin Zhang, Xiaohu Xie, Dingding Zhuang, Wenwen Shen, Huifen Liu, Wenhua Zhou

**Affiliations:** ^1^Laboratory of Behavioral Neuroscience, Key Laboratory of Addiction Research of Zhejiang Province, School of Medicine, Ningbo Institute of Microcirculation and Henbane, Ningbo Kangning Hospital, Ningbo University, Ningbo, China; ^2^Molecular Diagnostic Laboratory, Ningbo Institute of Medical Science, The Affiliated Hospital of School of Medicine, Ningbo University, Ningbo, China; ^3^Department of Psychiatry, Ningbo Kangning Hospital, Ningbo, China

**Keywords:** heroin, drug abuse, biomarker, plasma microRNA, addiction

## Abstract

Heroin use disorder is a chronic and relapsing disease that induces persistent changes in the brain. The diagnoses of heroin use disorders are mainly based on subjective reports and no valid biomarkers available. Recent researches have revealed that circulating miRNAs are useful non-invasive biomarkers for diagnosing brain diseases such as Alzheimer's disease, multiple sclerosis, schizophrenia, and bipolar disorder. However, studies on circulating miRNAs for the diagnosis of heroin use disorders are rarely reported. In this study, we investigated the differential expression of plasma miRNAs in 57 heroin-dependent patients. Based on literature research and microarray analysis, two candidate miRNAs, miR-320a and let-7b-5p, were selected and analyzed by quantitative real-time RT-PCR. The results showed miR-320a and let-7b were significantly upregulated in plasma of the heroin-dependent patients compared to that in healthy controls. The area under curves (AUCs) of receiver operating characteristic (ROC) curves of miR-320a and let-7b-5p were 0.748 and 0.758, respectively. The sensitivities of miR-320a and let-7b-5p were 71.9 and 70.2%, while the specificities of miR-320a and let-7b-5p were 76.1 and 78.3%, respectively. The combination of these two miRNAs predicted heron dependence with an AUC of 0.782 (95% CI 0.687–0.876), with 73.7% sensitivity and 82.6% specificity. Our findings suggest a potential use for circulating miRNAs as biomarkers for the diagnosis of heroin abuse.

## Introduction

Heroin, a morphine derivative, is one of the most powerfully addictive drugs abused in the world. People who abused heroin are among the most marginalized and disadvantaged drug users. They experience poor health outcomes with a greater chance of premature death, increased risk of both non-fatal and fatal drug overdoses, and high rates of potentially life-threatening infectious diseases, such as tuberculosis, hepatitis, and HIV. Heroin addiction is a relapsing and chronic brain disorder that causes persistent alterations in synaptic plasticity (1–3). However, the underlying molecular mechanisms that lead to addiction remain poorly understood. Screening and confirmation of drug dependence or use disorders are mainly carried out by analyzing drugs and their metabolites in blood samples, which are not stable and at ever decreasing levels (4). Moreover, the diagnoses of the severity of drug use disorders are mainly based on subjective reports, and there are no objective biomarkers available. Therefore, identifying stable and objective biomarkers for drug abuse is of crucial importance.

MicroRNAs (miRNAs) are a class of stable non-coding small RNA molecules able to regulate gene expression by binding to 3'-UTR regions of their mRNAs targets (5). MiRNAs are particularly abundant in the central nervous system, specific miRNAs whose expression is altered are identified to regulating the behavioral effects of addictive drugs (6–9). Recent studies of the association between miRNA dysfunction and addiction have shown that miRNAs are involved in substance use disorders such as alcohol, cocaine, and amphetamine addiction (9–11). MiRNAs play key roles in the development of addiction by directly regulating synaptic remodeling, dendritic spine morphogenesis, rewarding properties of drugs, and drug-seeking behavior (3). Moreover, researches showed levels of specific miRNAs that protect against drug addiction were elevated in the brain (12). Chronic treatment of morphine upregulated the expression levels of miRNA-23b and let-7 miRNAs, which in turn repress mu-opioid receptor (MOR1) mRNA at the post-transcriptional level (13). As such, miRNAs represent promising biomarkers that integrate gene expression and regulation with temporally changing substance use disorders processes.

Identification of addiction-related human-specific circulating miRNAs will be of important clinical significance. Recent studies have shown that heroin exposure upregulated a panel of plasma miRNAs related to immunity and virus infection (14, 15). Previous studies have shown that there are a large number of stable miRNAs in plasma and serum, and some of these miRNAs may be used as biomarkers for mental health diseases such as schizophrenia, bipolar disorder, etc (16). However, whether circulating miRNAs can be potential biomarkers of heroin addiction is still not well-studied. Drug abuse can cause detrimental impacts on brain cells, contributing to neuronal cell apoptosis and neurodegeneration (1). Moreover, drug abuse can also cause blood-brain barrier dysfunction (17). Multiple studies reported that plasma proteins and miRNAs were significantly associated with brain atrophy related to Alzheimer's disease, indicating they can act as surrogate biomarkers for events in the brain (18, 19). Thus, we hypothesized that circulating miRNAs are associated with brain miRNA, and upregulated brain miRNAs resulted from drug addiction can be secreted into the bloodstream and increase the level of specific miRNAs in plasma. Therefore, it is was quite meaningful to studying these kinds of circulating miRNAs, which have the potential to be non-invasive biomarkers for diagnosing drug addictions and appraising their severity.

Our work is to study the plasma expression pattern of specific miRNAs highly related to drug addiction, which is quite important for the diagnosis and treatment of this disorder. To clarify certain microRNA expression levels in humans and their relationships with heroin use, plasma samples from 4 heroin-dependent patients and 5 normal healthy controls were collected and screened by genome-wide profiling microarray analysis, two distinctly expressed plasma miRNA let-7b-5p and miR-320a were identified. Then, we measured them in the 57 cases of heroin-dependent patients.

## Materials and Methods

### Study Subjects

**Figure 1:**
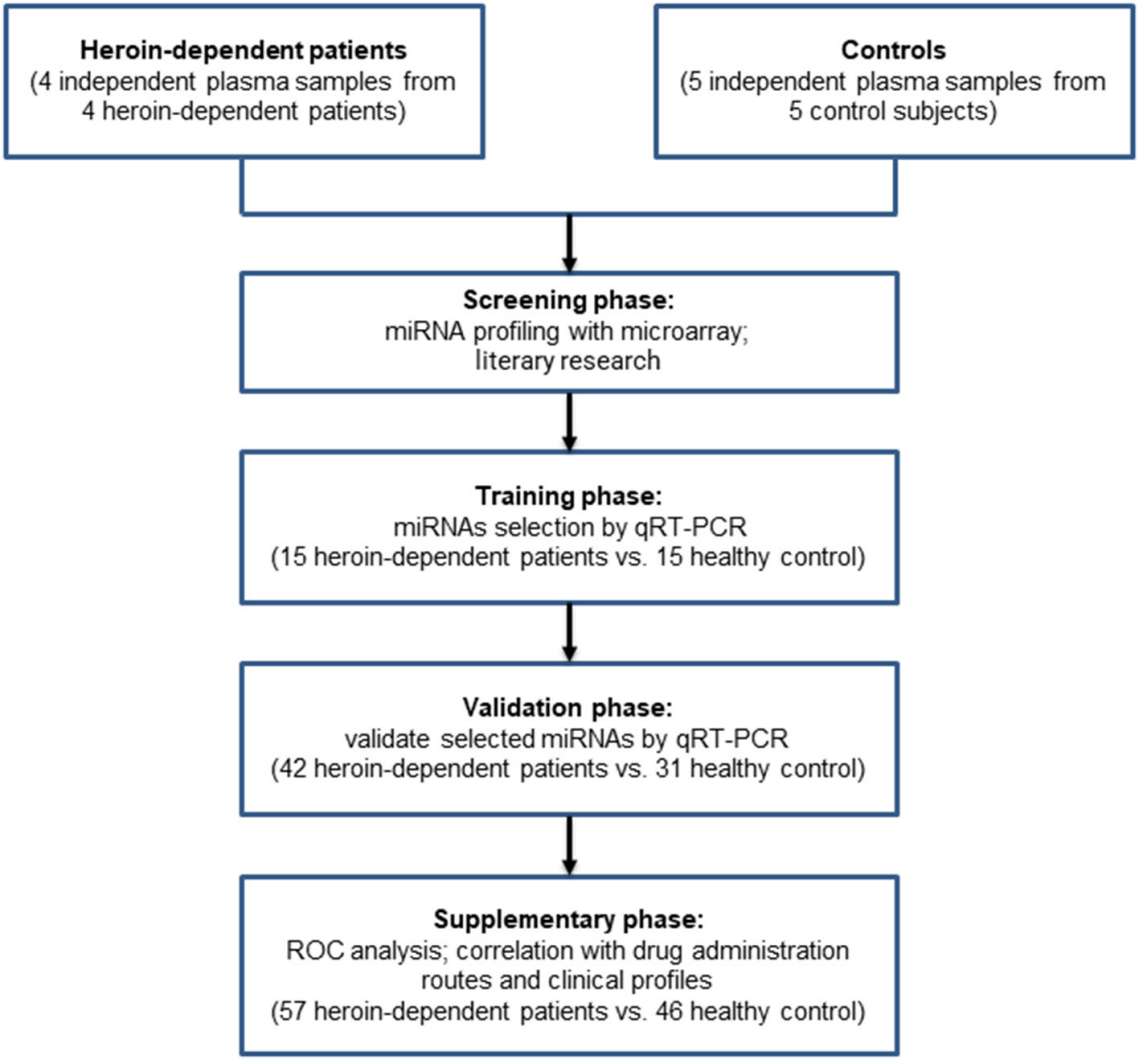
Flowchart of the study design.

A total of 61 male heroin-dependent patients (case group) were recruited from Ningbo city through the Ningbo Addiction Research and Treatment Center of Zhejiang Province, the People's Republic of China from June 2014 to June 2018, and performed through the Structured Clinical Interview of the Diagnostic and Statistical Manual of Mental Disorders, Fourth Edition (DSM-IV). The general demographic data of these subjects were collected. We also collected detailed information about drug use history (such as use methods, years of drug use, and daily dose) as well as the smoking habit by interview. We did not include female subjects in this study because there were fewer female heroin-dependent patients. All subjects were males of Han-Chinese origin and had no other psychiatric disorders such as schizophrenia, bipolar disorder, major depressive disorder, anxiety disorder, or a history of chronic systemic illness such as cardiac, renal, pulmonary, hepatic, endocrine, metabolic, and autoimmune disorders, or a history of chronic pain problem treated with opioid analgesics. The heroin-dependent patients then had urine tests screened for heroin metabolites, methamphetamine, cocaine, or ketamine. All subjects were morphine positive and had no other illicit drug positive except heroin or tobacco. A total of 51 healthy individuals (control group) were recruited from among voluntary blood donors in the Ningbo Blood Center of Zhejiang Province. The general demographic data and smoking habits were also recorded and documented. A multi-stage, case-control study was designed to identify a panel of plasma miRNAs related to heroin dependence. The characteristics of the study subjects were summarized in Table 1. The whole study was composed of four phases: screening phase, training phase, validation phase, and supplementary phase. The work flowchart of the study is shown in Figure 1. For the training group, participants from all over China were recruited from the inpatient department. While for the validate group, the participants were recruited from the methadone clinic, most of them were residents of Zhejiang province. The study protocol was approved by the Ethics Committee of Ningbo Addiction Research and Treatment Center, and written informed consent was obtained from all subjects.

**Table 1:** The demographic characteristics of the participants.

**Characteristics**	**Heroin dependent patients**	**Healthy controls**	***P*-value**
Ethnicity: Chinese Han *n* (%)	61 (100)	51 (100)	1^a^
Sex: male *n* (%)	61 (100)	51 (100)	1^a^
Age (years): mean (SD)	35.7 (6.8)	34.8 (6.)	0.416^a^
**Marital status:** ***n*** **(%)**			0.012^b^
Married	42 (70.5)	46 (90.2)	
Single	15 (23.0)	5 (9.8)	
Divorced	4 (6.5)	0	
**Drug use history**			
Heroin addiction time (years): mean (SD)	9.7 (6.7)	0	
Onset age of drug use (years): mean (SD)	25.9 (7.1)	None	
**Education:** ***n*** **(%)**			<0.001^c^
Primary	2 (3.3)	3 (6.5)	
Middle school	50 (82.0)	6 (6.5)	
High school	8 (13.1)	8 (15.2)	
College	1 (1.6)	34 (71.8)	
**Employment:** ***n*** **(%)**			<0.001^b^
Self employed	44 (71.7)	15 (29.4)	
Unemployed	14 (23.3)	0	
Employed	3 (5)	36 (70.6)	
**Drug taking mode:** ***n*** **(%)**			
Smoking	41 (67.2)	0	
Injection	20 (32.8)	0	
Daily average amount (grams): mean (SD)	0.84 (0.57)	0	

a *Student t-test;*

b *Fisher's exact test;*

c *Pearson's chi-square test*.

### Blood Collection and RNA Extraction

The venous blood samples of all subjects were obtained after overnight fasting (for at least 8 h) in the morning that were demonstrated to not significantly affect the overall circulating miRNA profiles (20). For heroin-dependent patients, blood was drawn within 1 week since the last heroin consumption. Two milliliter peripheral blood was drawn from participants into tubes containing EDTA. Then, within 30 min after blood collection, blood samples were centrifuged at 1,200 g for 12 min at room temperature. The supernatant plasma was then transferred into new microcentrifuge tubes and centrifuged at 12,000 g for 15 min at 4°C. After removing cellular debris, purified plasma was stored as single-use aliquots at −80°C until use. When extraction, the miRNeasy Serum/Plasma Kit (Qiagen, Germany) was used to purify the miRNA from 200 μl plasma according to the manufacturer's specifications. Briefly, plasma was mixed thoroughly with 1 ml of Qiazol then supplemented with 3.5 μl miRNeasy Serum/Plasma Spike-In Control (1.6 ×10^8^ copies/μl working solution). RNA is eluted in 13 μl of nuclease-free water. The concentration of RNA was measured using a NanoDrop ND-2000 (Thermo Scientific).

### MicroRNA Microarray Expression Profiling

Plasma miRNAs profilings of 5 healthy controls and 4 heroin-dependent patients were determined using microRNA microarray [Agilent Human miRNA (8^*^60K) V19.0 array] which containing probes for 2006 human microRNAs. The NanoDrop ND-2000 (Thermo Scientific) was used to quantify total RNA. And Agilent Bioanalyzer 2100 (Agilent) was used to assess RNA integrity. Sample labeling, microarray hybridization, and washing were implemented according to the manufacturer's instructions. Briefly, total miRNA was dephosphorylated and denatured, followed by pCp-Cy3 labeling. After purification, labeled RNAs were hybridized onto the microarray according to the array manual. After washing, the slides were scanned into image files with the Agilent Scanner G2505C (Agilent Technologies) and the scanned images were analyzed using Agilent Feature Extraction Software (Agilent Technologies). The resulting data have been deposited in NCBI's Gene Expression Omnibus (GSE160275).

### Quantitative Real-Time RT-PCR (qRT-PCR) for Candidate miRNAs

Total miRNA was transcribed to cDNA using the miScript II RT Kit (Qiagen, Germany). Diluted cDNA (1:10) was used for detecting miRNA expression by Q-PCR using the miScript SYBR Green PCR Kit with miScript Primer Assay (Qiagen, Germany). All reactions were run in triplicate, and results were normalized to miR-16, a commonly used endogenous reference gene. For further data analysis, only those miRNAs with a CT value equal to or below 30, a cut-off recommended by the manufacturer (Qiagen, Germany), were taken into account. Reactions without reverse transcription or RNA templates were used as a negative control. First, ΔCt was calculated by subtracting the Ct values of the miRNA of interest from miR-16. Then, the relative expression data of miRNAs were calculated using the 2^−Δ*Ct*^ method and subjected to base-10 logarithmic transformation (log_10_2^−Δ*Ct*^).

### Statistical Analyses

All data analysis was performed using GraphPad Prism 8 (GraphPad Software Inc., San Diego, CA, USA) except Chi-square test, Fisher's exact test, and Spearman's rank correlation analysis, which were analyzed by the SPSS 19.0 (IBM, USA). Enumeration data were indicated as percentages, whereas measurement data were expressed as mean ± standard error of the mean (SEM). Normality analysis of each group was performed by using the D'Agostino & Pearson test. For comparing two independent groups, the statistical significance was evaluated by unpaired *t*-test with Welch's correction when the variables are unequal variance and follow a normal distribution, non-parametric Mann-Whitney *U*-test when the variables were abnormally distributed. For comparing multiple groups, the statistical significance of normally distributed variables with unequal variances was assessed by Welch's ANOVA test followed by Games-Howell *post-hoc* test for multiple comparisons, and abnormally distributed variables were determined by the Kruskal–Wallis test followed by Dunn's multiple comparisons *post hoc* test. Differences between the means were considered statistically significant at *p*-value < 0.05 (two-tailed). For analyzing effect size, different approaches were used. For the independent samples *t*-test with different standard deviations, Hedges' g is used to make a statement about the effect size. For non-parametric Mann-Whitney *U*-test, r [r = z/sqrt(N)] is used. Values range between 0.5 and 0.8 were regarded as an intermediate effect, while ≥0.8 was regarded as a large effect (21). ROC curves and AUCs were established to evaluate the diagnostic value of let-7b-5p and miR-320a for distinguishing heroin-dependent patients from healthy controls. Demographic variables were compared between patients and healthy controls with the Chi-square test or the Fisher's exact test for qualitative variables and a *t*-test for quantitative variables. Correlations between miRNA profiles and clinical profiles of heroin-dependent patients were determined by Spearman's rank correlation analysis.

## Results

### The Demographic Characteristics of the Participants

The clinical characteristics of the participants are shown in Table 1. There were 61 heroin-dependent patients and 51 healthy controls in our study. No significant differences were found between groups in age and gender (*p* > 0.05). The average age of the heroin group was 35.7 years and had used heroin for an average of 9.7 years. Forty-one participants of the heroin group use heroin by smoking while 20 by injection. The heroin group had a significantly lower level of education than the healthy control group (*p* < 0.001).

### Candidate miRNAs From Microarray Screen

**Figure 2:**
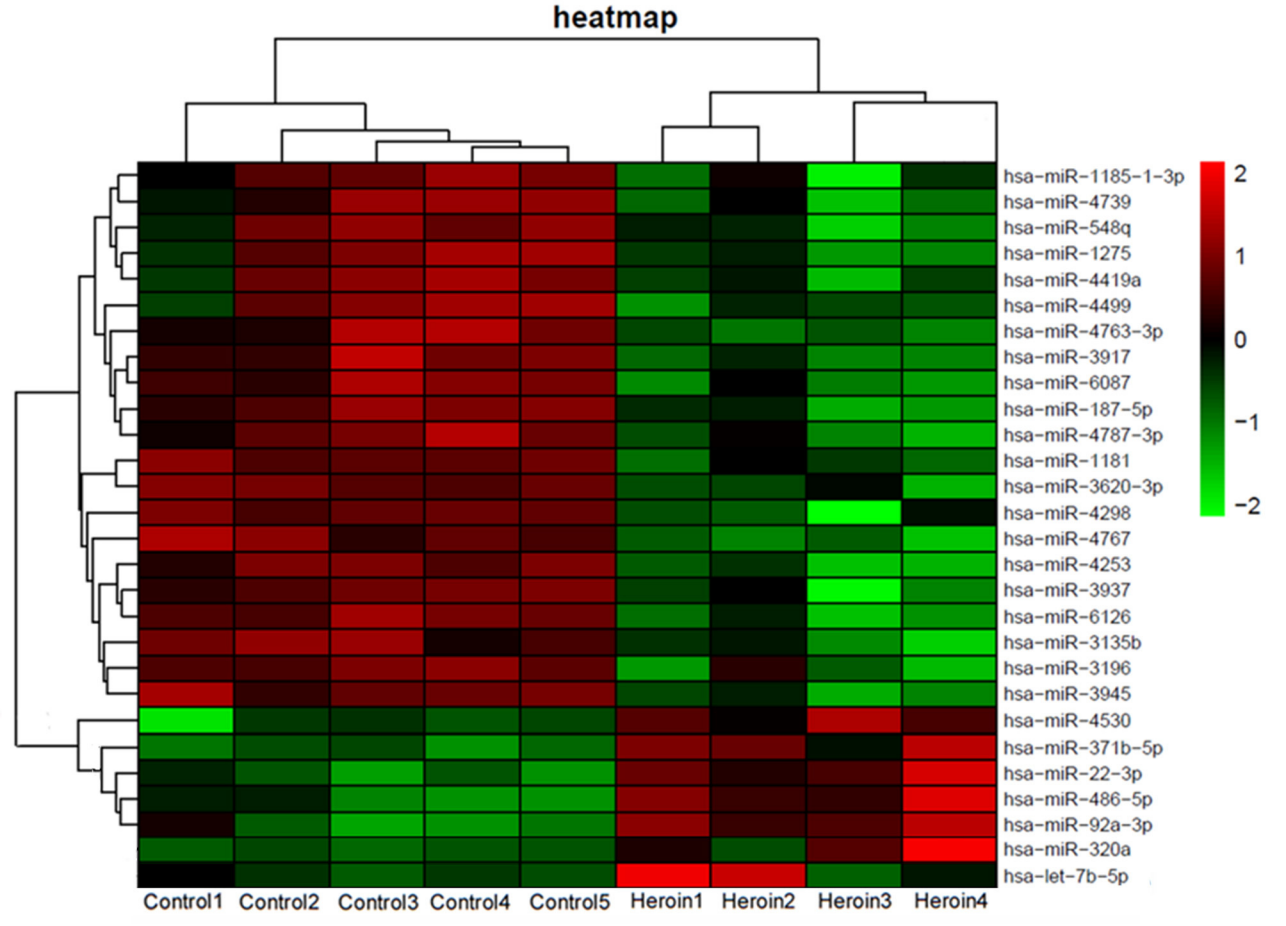
Heat map of miRNA microarray expression data from plasma samples of heroin-dependent patients (*n* = 4) and healthy control subjects (*n* = 5). MiRNA expression is hierarchically clustered on the y-axis. Each column represents a sample, while each row represents a miRNA. The legend on the right indicates the miRNA represented in the corresponding row. The relative miRNA expression is depicted according to the color scale shown on the right. Red indicates upregulation; and green, downregulation.

To determine the differential miRNA levels in heroin-dependent patients, we comparatively profiled plasma miRNA expression of four heroin-dependent patients and five healthy controls using Agilent Human miRNA (8^*^60 K) V19.0 Array. The microarray screen result showed that plasma miRNAs differed significantly among the two groups. MiRNAs showing a mean fold-change >2.0 and *p*-value < 0.05 were illustrated in the heat map generated from the unsupervised clustering analysis (Figure 2), while the *p*-value, foldchange, and false discovery rate (FDR) of these distinct miRNAs were shown in Supplementary Table 1.

### Differential Expression of Candidate Plasma miRNAs in Heroin Subjects

Drug abuse can cause detrimental impacts on brain cells, which results in increased expression of specific miRNAs that protect against drug addiction (12). Moreover, drug abuse can also lead to blood-brain barrier dysfunction (17). Therefore, we hypothesized that upregulated miRNAs of the brain resulted from drug addiction may enter the blood and elevate the level of specific miRNA in plasma, which may reflect the severity of drug addiction. Literature analysis showed levels of two brain-expressed miRNA, miR-320a and let-7b-5p, were increased during neuronal damage and differentiation in the brain (22–24). And our miRNA microarray results also showed plasma levels of miR-320a and let-7b-5p were elevated with a fold-change of 4.0 and 1.4, respectively, in heroin-dependent patients (Figure 2). Thus, we selected these two miRNAs for further analysis. Further analyzing the expression pattern of the two miRNAs by microarray assay showed miR-320a was also upregulated in methamphetamine patients, while let-7b-5p showed no difference (Supplementary Figure 1).

**Figure 3:**
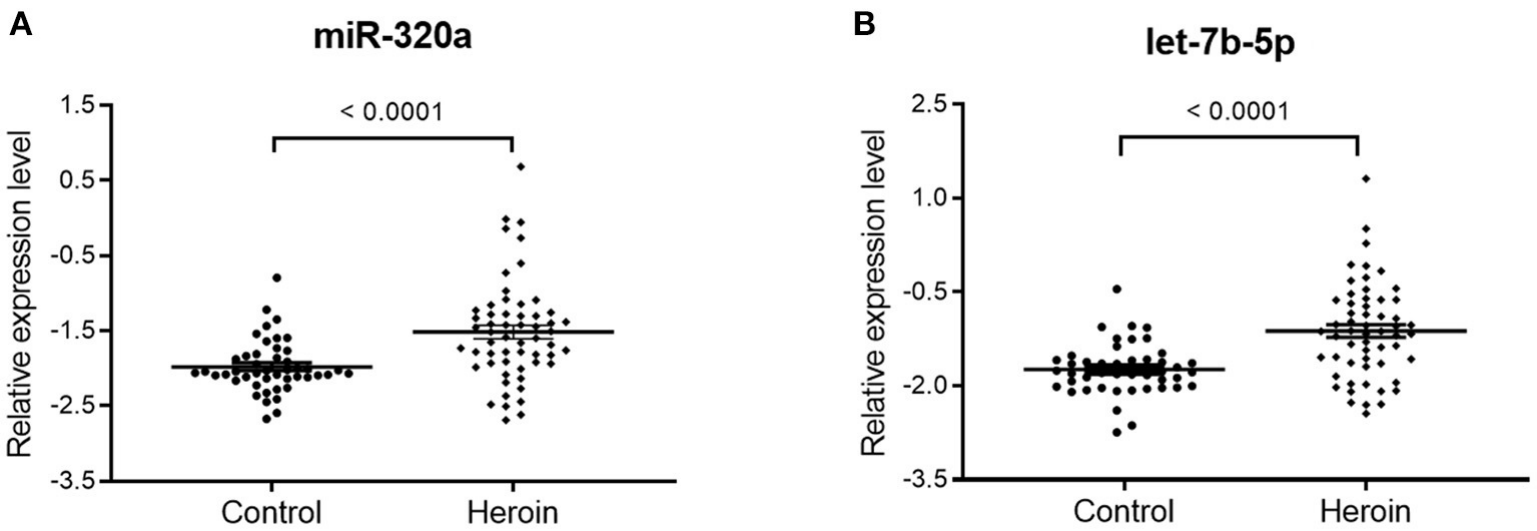
The differential expression of miR320a and let-7b-5p between heroin-dependent patients and control subjects. A total of 57 heroin-dependent patients and 46 healthy controls in the training and validation sets were used to analyze the difference. **(A)** miR-320a (Mann-Whitney *U*-test; Control group vs. heroin group, *p* < 0.0001); **(B)** Let-7b-5p (unpaired *t*-test; Control group vs. heroin group, *p* < 0.0001). Data are shown as scatter plots. Mean values are indicated by horizontal bars.

qRT-PCR assay was used to confirm the expression level of the selected miRNAs. The study participants were divided randomly into two sets: a training set and a validation set. The demographic information for the two sets was showed in Supplementary Table 2. In the training set, 15 heroin-dependent patients and 15 controls were examined by qRT-PCR. The results showed the plasma levels of miR-320a and let-7b in patients were significantly higher than in healthy controls (*p* < 0.05, Table 2). The changes in the levels of miR-320a and let-7b were further verified by qRT-PCR in another cohort of samples in the validation set, which included 42 heroin-dependent patients and 31 matched controls. Consistent with the results from the training set, the plasma levels of miR-320a and let-7b-5p were significantly increased in the heroin group (*p* < 0.05, Table 2). Then, the participants enrolled in the training and validation sets were combined into one cohort, and differences in plasma levels for the two miRNAs in total participants were analyzed and shown in Figure 3. Both miRNAs showed significantly increased expression patterns in heroin-dependent patients (*P* < 0.0001). Calculating the effect sizes showed heroin dependence had a large effect on plasma miR-320a (*r* = 0.94) and let-7b-5p (Hedges' g = 1.072) level.

**Table 2:** Relative expression levels of two plasma miRNAs in the training and validation sets^a^.

**miRNA**	**Training set**			**Validation set**		
	**Controls (*n* = 15)**	**Heroin dependents (*n* = 15)**	***P*-value**	**Controls (*n* = 31)**	**Heroin dependents (*n* = 42)**	***P*-value**
miR-320a	−2.02 ± 0.09	−1.61 ± 0.16	0.038^b^	−1.96 ± 0.06	−1.48 ± 0.11	0.0001^c^
let-7b-5p	−1.86 ± 0.12	−1.17 ± 0.14	0.001^b^	−1.68 ± 0.07	−1.11 ± 0.13	0.001^c^

a *miRNAs relative plasma levels are presented as Mean ± SEM;*

b *Student t-test;*

c *Nonparametric Mann-Whitney U-test*.

### ROC Analysis for Differential Expression of Circulating miRNAs

**Figure 4:**
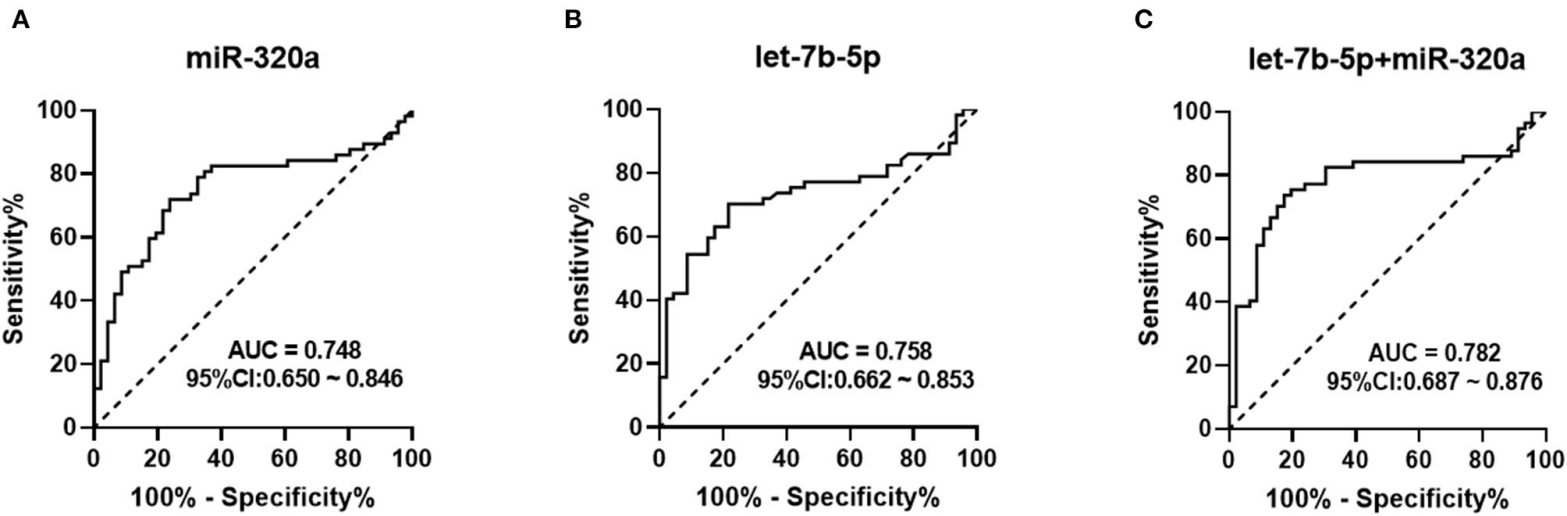
Receiver Operation Characteristic (ROC) curve for the ability of miR-320a and let-7b-5p to distinguish heroin-dependent patients from healthy controls. AUC is the area under receiver-operator characteristic curve. The diagonal line represents a reference line of zero sensitivity and zero specificity. **(A)** ROC curve for miR-320a to discriminate heroin-dependent patients from healthy controls (AUC of 0.748, sensitivity = 71.9%, specificity = 76.1%, *p* < 0.0001); **(B)** ROC curve for let-7b-5p to discriminate heroin-dependent patients from healthy controls (AUC of 0.758, sensitivity = 70.2%, specificity = 78.3%, *p* < 0.0001); **(C)** ROC curve for a combination of let-7b-5p and miR-320a to discriminate heroin-dependent patients from healthy controls (AUC of 0.782, sensitivity = 73.7%, specificity = 82.6%, *p* < 0.0001).

ROC curve analyses were employed to evaluate the predictive power of circulating miRNAs for differentiating between the patients and healthy controls. The combined cohort of the training and validation sets that consisted of 57 heroin-dependent patients and 46 matched controls were used for analysis. Both miR-320a and let-7b-5p turned out to be significant predictors. The AUC of miR-320a was 0.748 (95% CI: 0.650–0.846; *p* < 0.0001) with a cut-off value of −1.829. A sensitivity of 71.9% and a specificity of 76.1% were achieved for identifying heroin addiction (Figure 4A); The AUC of let-7b-5p was 0.758 (95 % CI: 0.662–0.853; *p* < 0.0001) with a cut-off value of −1.573. A sensitivity of 70.2% and a specificity of 78.3% were achieved for identifying heroin addiction (Figure 4B). Then, we determined whether the combination of miR-320a and let-7b-5p could provide a more effective screening for heroin dependence. As shown in Figure 4C, the combination of the two miRNAs yielded an AUC value of 0.782 (95% CI, 0.662–0.854; *P* < 0.0001) with 73.7% sensitivity and 82.6% specificity in distinguishing heroin-dependent patients from healthy subjects. We can see that the discrimination power of the two miRNAs combined was superior to that of a single miRNA signature.

### Correlation of Plasma miRNA Level With Drug Administration Routes and Clinical Profiles

**Figure 5:**
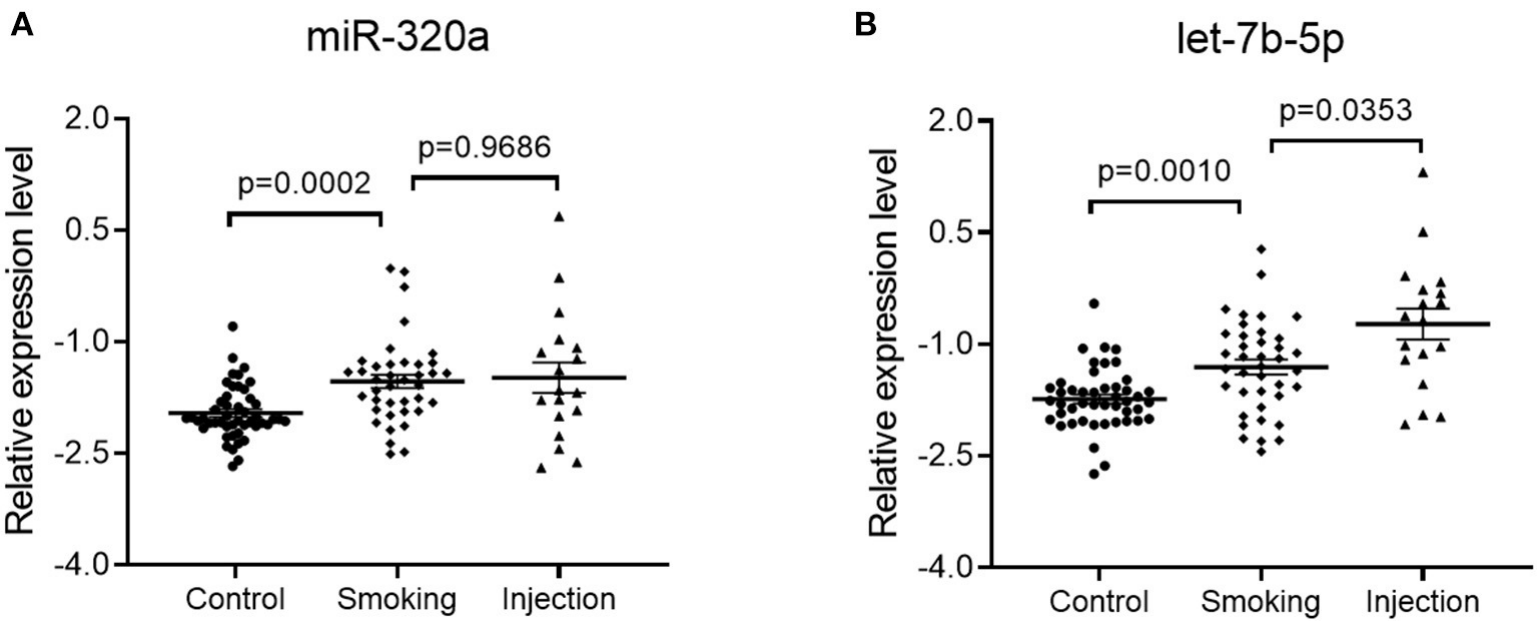
Impact of administration routes on the plasma miRNA expression level. Plasma specimens were collected from healthy controls (*n* = 46), heroin smoking patients (*n* = 39), and heroin injection patients (*n* = 18) enrolled in the training and validation sets. Statistical significance was calculated by Welch's ANOVA test followed by a Games-Howell *post-hoc* test for multiple comparisons. **(A)** Relative expression levels of miR-320a; Control group vs. smoking group, adjusted *p*-value = 0.0002; Smoking group vs. injection group, adjusted *p*-value = 0.9686; **(B)** Relative expression levels of let-7b-5p; Control group vs. smoking group, adjusted *p*-value = 0.001; Smoking group vs. injection group, adjusted *p*-value = 0.0353. Data are shown as scatter plots. Mean values are indicated by horizontal bars.

To investigate whether administration routes affect the expression pattern of plasma miRNA, total heroin-dependent patients of training and validation set were classified into two groups, the heroin injection group (*n* = 39) and the heroin smoking group (*n* = 18). As shown in Figure 5B, the levels of plasma let-7b-5p were significantly upregulated in the heroin injection group, while miR-320a showed no significant difference (Figure 5A). Moreover, levels of plasma miR320a and let-7b-5p were significantly higher in heroin smokers when compared to the healthy control group (Figures 5A,B). After that, we examined whether the dysregulated plasma miRNAs are associated with the clinical profiles of heroin-dependent patients. No marked association was observed between the plasma levels of the two miRNAs and age, onset age of drug use, dosages per day, times of drug-using per day, drug abuse time, and other baseline characteristics (all *p*-value > 0.05) (Supplementary Table 3).

To further investigated whether tobacco using has any impact on the plasma miRNA expression pattern, our total healthy participants enrolled in the training and validation sets were classified into the non-smoker group (*n* = 15) and smoker group (*n* = 31). All heroin-dependent patients enrolled in our study are also tobacco users and were classified into one group (*n* = 57). As shown in Supplementary Figure 2, plasma levels of let-7b-5p and miR-320a were not significantly different between the healthy non-smoker group and healthy smoker group (*p*-value > 0.05), while the levels of both miRNAs were found to be significantly increased in the heroin-dependent patient group.

## Discussion

At present, no effective treatment or clinical diagnosis has been developed for heroin dependence, which is a chronic and relapsing disorder that induces persistent changes in the brain (1). Recent studies showed circulating miRNAs in plasma are found to be stable and readily detectable by RT-PCR, which are attractive candidates for diagnosis, prognosis, and targets for therapy in a variety of disease settings (25, 26). However, it was not well-understood whether or not circulating miRNAs can be used to reflect drug addiction status.

MiRNAs are abundantly expressed in the nervous system, suggesting they may be particularly important for neural development and function (27). Recently, there has been ample evidence for the important role of miRNAs in drug addiction. For instance, chronic morphine treatment increases let-7 and miRNA23b expression in a time- and dose-dependent manner and suppresses the association of MOR1 mRNA with polysomes (8, 13, 28). Expression of miR-24, miR-127, miR-186, and miR-222 was upregulated in the prefrontal cortex region of rats with escalated methamphetamine use (29). These findings suggested chronic exposure to addictive drugs may significantly alter brain miRNA profiles. Moreover, accumulating evidence also suggests that circulating miRNAs can be used as biomarkers for brain diseases, such as cerebral ischemia, psychiatric disorders, and neurodegenerative disorders (19, 25, 30, 31). However, whether circulating miRNAs can be biomarkers for heroin addiction is not well-studied, and only a few preliminary studies available. Heroin use can upregulate a panel of plasma exosomal miRNAs associated with neuroinflammation and neurotoxicity (32). And recent studies have reported peripheral miRNAs, including miR-486-5p, miR-206, and let-7b-5p were dysregulated in patients with heroin use disorder (33). However, due to the limited number of subjects and unbalanced sample size, further investigations are necessary to confirm the clinical significance of these findings. In the present study, both our miRNA array analysis and qRT-PCR results demonstrated that plasma miRNAs were dysregulated in heroin-dependent patients, supporting the notion that heroin abuse has a significant impact on plasma miRNA expression level.

MiR-320a, located on chromosome 8, was previously suggested to be involved in depression, schizophrenia, cerebral ischemia, and neurodegenerative diseases (22–24). Circulating miRNA-320a has been reported to be highly expressed in coronary artery disease and arrhythmogenic cardiomyopathy patients (34). Coincidentally, repeated heroin use can cause serious cerebral vascular injury, arrhythmia, and pathological phenotypes similar to common neurodegenerative diseases (35–37). All these findings suggest our novel finding of an elevated level of circulating miR-320a most likely reflects the corresponding dysfunction in the brain caused by heroin addiction. In addition to statistical significance, analysis of the effect size showed heroin dependence had a large effect on the plasma level of miR-320a. Furthermore, analyzing the ROC curve of miR-320a indicates that peripheral miR-320a could distinguish heroin-dependent patients from healthy controls with high sensitivity and specificity. Therefore, miR-320a may have potential diagnostic value for heroin dependence. Since little is known about the relevance of miR-320a in heroin dependence, future studies are needed to determine the specific genetic networks or targets through which miR-320a affects pathways underlying heroin addiction.

Heroin and morphine both belong to the opioid class of drugs, and their euphoric effects are produced by their binding with MOR in the brain. A previous study showed MOR was a direct target of let-7. By binding to a let-7 binding site in its 3'UTR, let-7 can translocate and sequester MOR mRNA to P-bodies, leading to translation repression (28). Moreover, chronic morphine treatments cause a robust upregulation of let-7 expression in a mouse model of opioid tolerance (28). These findings suggest that let-7 plays an integral role in opioid tolerance, and increased expression of let-7 may reflect a higher level of tolerance as well as addiction severity. Let-7b-5p, a let-7 family miRNA, is a brain-specific miRNA that plays a significant role in neurogenesis and synapse formation (38). Its dysregulation is associated with schizophrenia, depression, neurodegenerative disorders, cerebral ischemia, and addiction (33, 39, 40). Here, our results indicated that let-7b-5p expression was also significantly upregulated in plasma of heroin-dependent patients, and calculation of the effect size showed heroin dependence had a large effect on the plasma level of let-7b-5p. The increased expression of let-7b-5p observed most likely reflects a higher level of heroin tolerance as well as addiction severity. Moreover, ROC analysis showed let-7b-5p has a significant diagnostic value for heroin dependence and yields an AUC of 0.758. Furthermore, plasma let-7b-5p level was also significantly higher in heroin injectors than in heroin smokers, indicating administration routes may have a significant impact on its expression. This result supports the generally believed notion that injectors had more severe withdrawal symptoms than smokers (41), and indicates a higher degree of severity of an individual's addiction (42), Thus, the upregulation of plasma let-7b-5p in heroin injectors most likely indicate higher severity of heroin dependence. It will be of interest to address, in future studies, any mechanistic role of let-7b-5p in heroin addiction pathogenesis, which may indicate the relevance and importance of let-7b-5p as possible targets for the treatment of drug addiction.

Studying the impact of potential confounding factors is quite important before miRNAs were used for diagnosing purposes. The use of tobacco is an important risk factor for the use of illicit drugs during adolescence (43). And an extremely high rate of tobacco co-use was observed in heroin-dependent patients (44). This may be due to opioids and tobacco similarly stimulate reward pathways including the dopaminergic, cannabinoid, and nicotinic acetylcholine systems (45). A previous study showed let-7d and miR-320 were upregulated in the smoking population, indicating smoking may be a confounding factor when using the expression of serum miRNAs for diagnosing pathological conditions (46). In this study, after compared healthy non-smokers with healthy smokers of our total healthy controls, we found the plasma level of miR320a and let-7b-5p showed no statistical difference, while they were significantly upregulated in the heroin-dependent patients. Thus, we think smoking may alter the expression of specific miRNAs but was not statistically significant in our experiment. Nevertheless, due to the extremely high rate of tobacco co-use, all heroin-dependent patients enrolled in our study are also tobacco users. Therefore, what effect does tobacco using has on the plasma level of these miRNAs in heroin-dependence patients is still unknown. Future studies to explore whether smoking can interfere with specific miRNA levels in heroin-dependent patients will help understand the relationship between these two types of addictions.

This study had several limitations. First, all patients in our study were males, so the same findings may not be extended to women. Second, miRNAs were reported to respond specifically to different drugs and thus regulate different pathways (3). Our preliminary miRNA microarray data also showed that the expression levels of miRNAs showed distinct patterns between methamphetamine-dependent patients and heroin-dependent patients. Thus, specific miRNAs may be involved in different types of drug dependence. And the effects of heroin dependence on the circulating miRNAs we found in this study may not extend to other drugs. Third, drug using disorders are complex disorders and often accompanied by neuropsychiatric disorders. Thus, the diagnosis of drug-using disorders is quite challenging. Though our study found miR-320a and let-7b-5p were distinctly expressed in heroin use disorder patients, the sensitivity and specificity of these miRNAs are not good enough for diagnosis purposes in clinical practice. Combined the two miRNAs seems to improve the diagnostic power, however, still failed to reach the required standard. Thus, future studies to find higher sensitivity and specificity biomarkers are still necessary. Fourth, we found heroin-dependent participants in our study have a significantly lower level of education compared to normal healthy controls. Measuring education levels against drug abuse is controversial. Multiple studies showed there is a strong association between a lower level of education and the risk of drug abuse (47). Nevertheless, low education level may also be associated with psychiatric disorders and/or socioeconomic status (48). Thus, different education levels may impact our study. Finally, we also analyzed the association between microRNAs expression and clinical profiles of heroin-dependent patients, which included the onset age of drug use, dosages per day, times of drug-using per day, and drug abuse time. Various evidence indicated that those profiles may be features of addiction severity. However, in our study, no significant association was observed between the levels of the two miRNAs and those clinical profiles. The negative results may be due to many reasons. One reason is the purity of drugs varies greatly, and clinical profiles are often considered inaccurate because they are collected by self-reporting (48). Besides, among heroin-dependent patients, the psychological and physical well-being of patients often varies greatly (49). Therefore, self-reported information is often not reliable, making the diagnosis of drug dependence or use disorders very difficult. In this respect, the development of new, specific, and objective diagnosis methods is quite important for the treatment of addiction.

In summary, our study systematically and comprehensively characterized the plasma miRNA expression patterns in heroin-dependent patients, demonstrates that plasma miR-320a and let-7b-5p showed distinct expression patterns in this type of patient. These miRNAs may have potential diagnostic value for heroin dependence. Further study of the relevance of miR-320a and let-7b-5p in heroin addiction may have significant implications for both uncovering the biological mechanism of heroin addiction and the development of new and more effective treatments.

## Data Availability Statement

The datasets generated for this study can be found in online repositories. The names of the repository/repositories and accession number(s) can be found in the article/Supplementary Material.

## Ethics Statement

The studies involving human participants were reviewed and approved by Ethics Committee of Ningbo Addiction Research and Treatment Center. The patients/participants provided their written informed consent to participate in this study.

## Author Contributions

HaL and WX designed and performed research, analyzing the data, and writing the paper. WX, JF, HM, JZ, XX, DZ, and WS performed research and data collection. WZ and HuL were responsible for the study concept and design, revising the paper, and organizing the discussion. All authors contributed to the article and approved the submitted version.

## Conflict of Interest

The authors declare that the research was conducted in the absence of any commercial or financial relationships that could be construed as a potential conflict of interest.

## Supplementary Material

Supplementary Figure 1 Heat map of miRNA microarray expression data from plasma samples of healthy controls (*n* = 5), heroin-dependent patients (*n* = 4), and methamphetamine-dependent patients (*n* = 5). MiRNA expression is hierarchically clustered on the y-axis. Each column represents an individual sample, while each row represents a miRNA. The legend on the right indicates the miRNA represented in the corresponding row. The relative miRNA expression is depicted according to the color scale shown on the right. Red indicates upregulation; and green, downregulation.

Supplementary Figure 2 Impact of tobacco using on the plasma miRNA expression level. Plasma specimens were collected from healthy non-smokers (*n* =15), healthy smokers (*n* = 31), and heroin-dependent patients (*n* = 57) enrolled in the training and validation sets. **(A)** Relative expression levels of miR-320a. Smoker group vs. non-smoker group, adjusted *p*-value > 0.9999; Smoker group vs. heroin-dependent group, adjusted *p*-value = 0.0002; Non-smoker group vs. heroin-dependent group, adjusted *p*-value = 0.0052; Statistical significance was calculated by Kruskal-Wallis test with Dunn's multiple comparisons test for multiple comparisons; **(B)** Relative expression levels of let-7b-5p. Smoker group vs. non-smoker group, adjusted *p*-value = 0.1944; Smoker group vs. heroin-dependent group, adjusted *p*-value < 0.0001. Statistical significance was calculated by Welch's ANOVA test with Games-Howell's multiple comparisons test for multiple comparisons. Data are shown as scatter plots. Mean values are indicated by horizontal bars.

Supplementary Table 1 Dys-regulated miRNAs in plasma of heroin-dependent patients.

Supplementary Table 2 Human subject characteristics of the training set and validation set^a^.

Supplementary Table 3 Correlations between the two altered plasma miRNAs' levels and clinical variables in heroin abusers.
