# Corrigendum: Increased Expression of Plasma miRNA-320a and let-7b-5p in Heroin-Dependent Patients and Its Clinical Significance

**DOI:** 10.3389/fpsyt.2021.733293

**Published:** 2021-07-26

**Authors:** Haixiong Liu, Wenjin Xu, Jiying Feng, Hong Ma, Jianbin Zhang, Xiaohu Xie, Dingding Zhuang, Wenwen Shen, Huifen Liu, Wenhua Zhou

**Affiliations:** ^1^Laboratory of Behavioral Neuroscience, Key Laboratory of Addiction Research of Zhejiang Province, School of Medicine, Ningbo Institute of Microcirculation and Henbane, Ningbo Kangning Hospital, Ningbo University, Ningbo, China; ^2^Molecular Diagnostic Laboratory, Ningbo Institute of Medical Science, The Affiliated Hospital of School of Medicine, Ningbo University, Ningbo, China; ^3^Department of Psychiatry, Ningbo Kangning Hospital, Ningbo, China

**Keywords:** heroin, drug abuse, biomarker, plasma microRNA, addiction

In the published article, there was an error in the affiliations and equal contributions in the author list.

The corrected affiliations and author list should be Haixiong Liu^1,2†^, Wenjin Xu^1†^, Jiying Feng^1^, Hong Ma^3^, Jianbin Zhang^1^, Xiaohu Xie^1^, Dingding Zhuang^1^, Wenwen Shen^1^, Huifen Liu^1*^ and Wenhua Zhou^1*^.

The authors apologize for this error and state that this does not change the scientific conclusions of the article in any way. The original article has been updated.

## Publisher's Note

All claims expressed in this article are solely those of the authors and do not necessarily represent those of their affiliated organizations, or those of the publisher, the editors and the reviewers. Any product that may be evaluated in this article, or claim that may be made by its manufacturer, is not guaranteed or endorsed by the publisher.

