# Supplementary figures and images for: Increased Expression of Plasma miRNA-320a and let-7b-5p in Heroin-Dependent Patients and Its Clinical Significance
Source: Front Psychiatry. 2021 Jun 29;12:679206. doi: 10.3389/fpsyt.2021.679206 (PMC8275879; doi:10.3389/fpsyt.2021.679206)

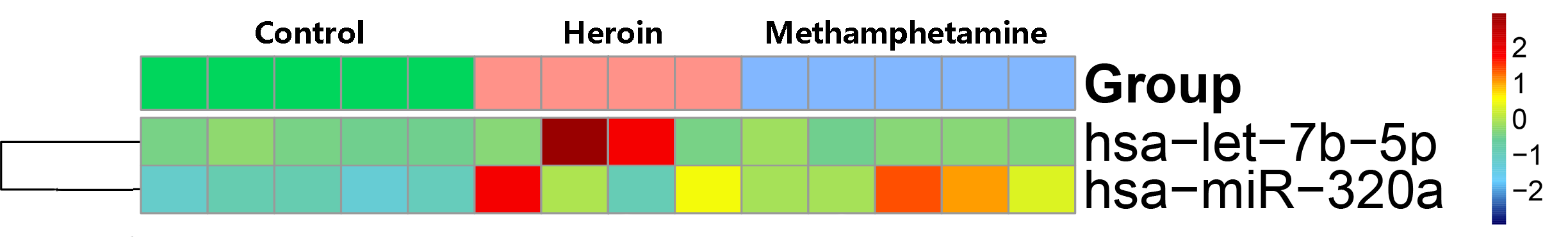

Supplement: Supplementary Figure 1 — Heat map of miRNA microarray expression data from plasma samples of healthy controls (n = 5), heroin-dependent patients (n = 4), and methamphetamine-dependent patients (n = 5). MiRNA expression is hierarchically clustered on the y-axis. Each column represents an individual sample, while each row represents a miRNA. The legend on the right indicates the miRNA represented in the corresponding row. The relative miRNA expression is depicted according to the color scale shown on the right. Red indicates upregulation; and green, downregulation. [file Image_1.TIF]

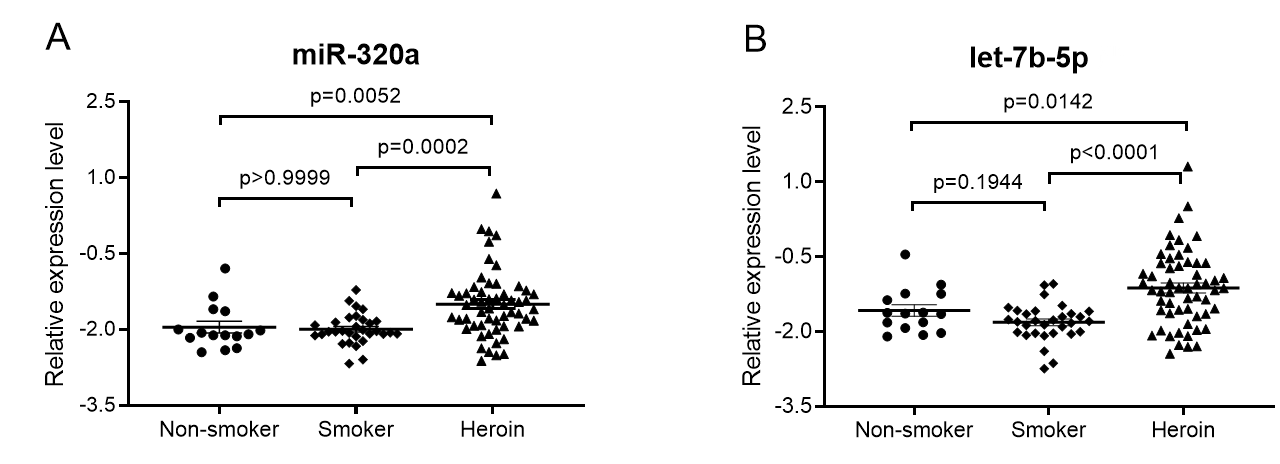

Supplement: Supplementary Figure 2 — Impact of tobacco using on the plasma miRNA expression level. Plasma specimens were collected from healthy non-smokers (n =15), healthy smokers (n = 31), and heroin-dependent patients (n = 57) enrolled in the training and validation sets. (A) Relative expression levels of miR-320a. Smoker group vs. non-smoker group, adjusted p-value > 0.9999; Smoker group vs. heroin-dependent group, adjusted p-value = 0.0002; Non-smoker group vs. heroin-dependent group, adjusted p-value = 0.0052; Statistical significance was calculated by Kruskal-Wallis test with Dunn's multiple comparisons test for multiple comparisons; (B) Relative expression levels of let-7b-5p. Smoker group vs. non-smoker group, adjusted p-value = 0.1944; Smoker group vs. heroin-dependent group, adjusted p-value < 0.0001. Statistical significance was calculated by Welch's ANOVA test with Games-Howell's multiple comparisons test for multiple comparisons. Data are shown as scatter plots. Mean values are indicated by horizontal bars. [file Image_2.TIF]
