# Supplementary material for: Increased Expression of Plasma miRNA-320a and let-7b-5p in Heroin-Dependent Patients and Its Clinical Significance
Source: Front Psychiatry. 2021 Jun 29;12:679206. doi: 10.3389/fpsyt.2021.679206 (PMC8275879; doi:10.3389/fpsyt.2021.679206)
Supplement: Supplementary Table 1 — Dys-regulated miRNAs in plasma of heroin-dependent patients. [file Table_1.DOCX]

**Table S1.** Dys-regulated miRNAs in plasma of heroin-dependent patients.

| Name | p-values | foldchange | FDR (False Discovery Rate) |
| --- | --- | --- | --- |
| hsa-miR-1181 | 0.004 | 0.479 | 0.342 |
| hsa-miR-1185-1-3p | 0.034 | 0.346 | 0.444 |
| hsa-miR-1275 | 0.007 | 0.395 | 0.342 |
| hsa-miR-187-5p | 0.005 | 0.304 | 0.136 |
| hsa-miR-22-3p | 0.005 | 5.871 | 0.136 |
| hsa-miR-3135b | 0.009 | 0.436 | 0.197 |
| hsa-miR-3196 | 0.026 | 0.495 | 0.342 |
| hsa-miR-3620-3p | 0.011 | 0.381 | 0.245 |
| hsa-miR-371b-5p | 0.010 | 2.442 | 0.181 |
| hsa-miR-3917 | 0.000 | 0.270 | 0.136 |
| hsa-miR-3937 | 0.026 | 0.314 | 0.450 |
| hsa-miR-3945 | 0.002 | 0.232 | 0.136 |
| hsa-miR-4253 | 0.003 | 0.295 | 0.371 |
| hsa-miR-4298 | 0.027 | 0.385 | 0.432 |
| hsa-miR-4419a | 0.012 | 0.343 | 0.371 |
| hsa-miR-4499 | 0.009 | 0.423 | 0.371 |
| hsa-miR-4530 | 0.006 | 2.393 | 0.245 |
| hsa-miR-4739 | 0.008 | 0.449 | 0.342 |
| hsa-miR-4763-3p | 0.002 | 0.499 | 0.245 |
| hsa-miR-4767 | 0.001 | 0.420 | 0.136 |
| hsa-miR-4787-3p | 0.008 | 0.263 | 0.181 |
| hsa-miR-486-5p | 0.005 | 6.566 | 0.342 |
| hsa-miR-548q | 0.012 | 0.349 | 0.342 |
| hsa-miR-6087 | 0.003 | 0.458 | 0.136 |
| hsa-miR-6126 | 0.004 | 0.231 | 0.136 |
| hsa-miR-92a-3p | 0.002 | 6.291 | 0.236 |
| hsa-let-7b-5p | 0.192 | 1.402 | 0.705 |
| hsa-miR-320a | 0.106 | 4.119 | 0.543 |
