# Supplementary material for: Increased Expression of Plasma miRNA-320a and let-7b-5p in Heroin-Dependent Patients and Its Clinical Significance
Source: Front Psychiatry. 2021 Jun 29;12:679206. doi: 10.3389/fpsyt.2021.679206 (PMC8275879; doi:10.3389/fpsyt.2021.679206)
Supplement: Supplementary Table 2 — Human subject characteristics of the training set and validation seta. [file Table_2.DOCX]

| **Table S2.** Human subject characteristics of the training set and validation set ^a^ | | | | | | | | | | |
| --- | --- | --- | --- | --- | --- | --- | --- | --- | --- | --- |
| **Characteristics** | **Training set** | | | |  | | **Validation set** | | | |
|  | **Controls (n = 15)** | | **Heroin group (n = 15)** | ***P-*value** | | **Controls (n = 31)** | | **Heroin group (n = 42)** | | ***P-*value** |
| **Age, years** | 32. 9± 6.7 | 33.4 ± 6.4 | | 0.454^b^ | | 35.7 ± 6.0 | | 36.3 ± 6.4 | 0.678^b^ | |
| Sex: male no (%) | 15 (100) | 15 (100) | | 1^d^ | | 31 (100) | | 42 (100) | 1^d^ | |
| **Marital status, no (%)** | 0.500^c^ 0.001^c^ | | | | | | | | | |
| Married | 12 (80.0) | 11 (73.3) | |  | | 31 (100) | | 29 (69.0) |  | |
| Single | 3 (20.0) | 4 (26.7) | |  | | 0 (0) | | 10 (23.8) |  | |
| Divorced | 0 (0) | 0 (0) | |  | | 0 (0) | | 3 (7.2) |  | |
| **Educational degree****, no (%)** | 0.002^c^  0.000^c^ | | | | | | | | | |
| Elementary School | 0 (0) | 0 (0) | |  | | 3 (9.7) | | 2 (4.8) |  | |
| Junior High School | 2 (13.3) | 11 (73.3) | |  | | 3 (9.7) | | 35 (83.3) |  | |
| Senior High School | 5 (33.3) | 3 (20) | |  | | 2 (6.4) | | 5 (11.9) |  | |
| College | 8 (53.4) | 1 (6.7) | |  | | 23 (74.2) | | 0 (0) |  | |
| **Drug use history** |  | | | | | | | | | |
| Onset age of drug use (years) | none | 25.6 ± 7.1 | |  | | none | | 25.7 ± 7.0 |  | |
| Drug use time (years) | 0 (0) | 7.8 ± 6.2 | |  | | 0 (0) | | 10.1 ± 6.7 |  | |
| Drug use dosages per day | 0 (0) | 0.90 ± 0.75 | |  | | 0 (0) | | 0.75 ± 0.45 |  | |
| Times of drug using per day | 0 (0) | 2.73 ± 0.96 | |  | | 0 (0) | | 3.0 ± 1.00 |  | |
| **Smoking status, no (%)** |  |  | | 0.008^c^ | |  | |  | 0.000^c^ | |
| Current smoker | 9 (60.0) | 15 (100) | |  | | 22 (70.1) | | 42 (100) |  | |
| Non-smoker | 6 (40.0) | 0 (0) | |  | | 9 (29.9) | | 0 (0) |  | |
| Past-smoker | 0 (0) | 0 (0) | |  | | 0 (0) | | 0 (0) |  | |
| **Drug manner, no (%)** |  | | | | | | | | | |
| Injection | 0 (0) | 10 (66.7) | |  | | 0 (0) | | 29 (69.0) |  | |
| Smoking | 0 (0) | 5 (33.3) | |  | | 0 (0) | | 13 (31.0) |  | |

^a^ Data are presented as mean ± SD. ^b^, Student t test; ^c^, Fisher’s exact test; ^d^, Pearson’s chi-square test.
