# Supplementary material for: Increased Expression of Plasma miRNA-320a and let-7b-5p in Heroin-Dependent Patients and Its Clinical Significance
Source: Front Psychiatry. 2021 Jun 29;12:679206. doi: 10.3389/fpsyt.2021.679206 (PMC8275879; doi:10.3389/fpsyt.2021.679206)
Supplement: Supplementary Table 3 — Correlations between the two altered plasma miRNAs' levels and clinical variables in heroin abusers. [file Table_3.DOCX]

| **Table S3.** **Correlations between the two altered plasma miRNAs’ levels and clinical variables in heroin abusers.** | | | | | | |  |
| --- | --- | --- | --- | --- | --- | --- | --- |
| **Variables** | | **Age** | **Onset age of drug use** | **Dosages per day** | **Times of drug using per day** | **Drug abuse time** | |
| **miR-320a** | *r_s_* | -0.030 | -0.076 | -0.132 | 0.284 | 0.157 | |
|  | *P*-Value | 0.824 | 0.573 | 0.328 | 0.144 | 0.976 | |
| **let-7b-5p** | *r_s_* | -0.107 | -0.056 | 0.059 | 0.149 | -0.031 | |
|  | *P*-Value | 0.429 | 0.677 | 0.664 | 0.268 | 0.817 | |
